# Analysis of epidemiological characteristics of extrapulmonary tuberculosis from South-Central China

**DOI:** 10.3389/fpubh.2024.1405358

**Published:** 2024-07-17

**Authors:** Yanyan Yu, Yu Xiang, Haican Liu, Shuliu Yang, Machao Li, Binbin Liu, Da Xu, Yaning Wu, Wenbin Li, Tanwei Fang, Jixiang Li, Donglei Xu, Kanglin Wan, Yunhong Tan, Xiuqin Yuan, Guilian Li

**Affiliations:** ^1^Clinical Laboratory, Hunan Provincial Tuberculosis Prevention and Control Institute & Hunan Chest Hospital, Changsha, Hunan, China; ^2^Institute of Reproduction and Stem Cell Engineering, School of Basic Medical Sciences, Central South University, Changsha, Hunan, China; ^3^National Key Laboratory of Intelligent Tracking and Forecasting for Infectious Diseases, Tuberculosis Reference Laboratory, National Institute for Communicable Disease Control and Prevention, Chinese Center for Disease Control and Prevention, Beijing, China; ^4^School of Public Health, University of South China, Hengyang, China

**Keywords:** extrapulmonary tuberculosis, prevalence, epidemiology, drug resistance, risk factors

## Abstract

**Objectives:**

This study aimed to investigate the epidemiological and drug resistance (DR) characteristics of extrapulmonary tuberculosis (EPTB) in South-Central China.

**Methods:**

EPTB inpatients who were culture-positive for *Mycobacterium tuberculosis* were retrospectively included in a study at a provincial TB hospital in Hunan, a province in South-Central China, from January 2013 to December 2021. Demographic, clinical, and drug susceptibility data were retrieved from TB treatment records. Descriptive statistical methods and a Chi-squared test were used to analyze the epidemiological and DR characteristics of EPTB patients. A logistic regression model was used to explore the risk factors of rifampicin-resistant/multidrug-resistant (RR/MDR)-EPTB.

**Results:**

A total of 1,324 cases were included. The majority of EPTB patients were in the age range of 20–29 years, were predominantly men (male-to-female ratio: 2.03), and were farmers (65.63%). Most EPTB cases were found in 2013 and 2017 from 2013 to 2021. The most prevalent subtypes of EPTB were lymphatic TB (29.83%, 395/1,324), multiple EPTB (20.85%, 276/1,324), and musculoskeletal TB (14.65%, 194/1,324). Musculoskeletal TB and genitourinary TB predominantly presented as exclusive EPTB forms, while lymphatic TB and pharyngeal/laryngeal TB often co-occurred with pulmonary TB (PTB). Drug susceptibility testing results showed that total DR rates (resistance to any of RFP, isoniazid [INH], streptomycin [STR], and/or ethambutol [EMB]) and RR/MDR rates in EPTB were 25.23% and 12.39%, respectively. Musculoskeletal TB exhibited the highest rates of total DR (31.40%), INH resistance (28.90%), STR resistance (20.10%), EMB resistance (6.20%), MDR (13.90%), and poly-DR (6.70%). The multivariable logistic regression model showed that patients aged from 20 to 59 years (compared to those aged 10 years), workers (compared to retirees), and EPTB patients from the south and west of Hunan (compared to those from the east of Hunan) were at an increased risk of developing RR/MDR EPTB (all *OR* values > 1).

**Conclusion:**

Our study provided a detailed account of the epidemiological and DR characteristics of EPTB in Hunan province, China. The significant DR rates, particularly in musculoskeletal TB cases, highlight the need for timely diagnosis, effective drug susceptibility testing, and the development of more effective treatment regimens for EPTB, especially targeting musculoskeletal TB treatments.

## Introduction

Tuberculosis (TB) remains a major public health problem, especially in developing countries. In 2022, 10.6 million people, including 5.8 million men, 3.5 million women, and 1.3 million children, were newly diagnosed with TB, and a total of 1.3 million people died from TB worldwide (1). TB typically affects the lungs (pulmonary TB, PTB), but it can also affect other sites (extrapulmonary TB, EPTB). The national TB prevention and control programs in many countries around the world mainly focus on PTB, while EPTB has been ignored to a certain extent (2). The World Health Organization (WHO) reported that EPTB represented 16% of the 7.1 million notified incident cases, ranging from 8% in the WHO Western Pacific Region to 24% in the Eastern Mediterranean Region in 2019 (3). EPTB accounts for an increasingly high proportion of TB. Multiple studies found that EPTB accounts for 11–33.4% of TB (4–7).

EPTB refers to tuberculous lesions that occur in organs and tissues other than the lungs. The most frequently involved organs or tissues are the lymph nodes, bones, joints, genitourinary, gastrointestinal, and central nervous systems (2). According to the lesion location of EPTB, it is difficult to diagnose on the basis of imaging characteristics and clinical symptoms, and a biopsy is required in many cases (8). Therefore, it is easy to misdiagnose EPTB as other diseases (9), which tends to underestimate the occurrence of EPTB. Several studies on the prevalence of EPTB have been reported (4–6). Li et al. (6) conducted a national cross-sectional study on EPTB with multistage, stratified cluster random sampling during 2020–2021. Kang et al. performed a large-scale multicenter observational study on EPTB in 21 hospitals in 15 provinces in China from 2011 to 2017 (4). Chu et al. (5) also reported the epidemiology of pediatric EPTB according to a national, multicenter study in China. These studies showed the epidemiological situations of EPTB nationally or outside of Hunan province, where little such data are known.

Hunan province, located in South-Central China, has a high prevalence of TB, with its number and incidence ranking second and fifth among the provinces of China in recent years (7). Hunan has achieved significant progress in TB control and reported a TB incidence of 66.4/100,000 in 2022, which is 18% less compared to 2018 (10). As part of addressing TB, the prevention and control of EPTB were also important. However, the epidemiological characteristics of EPTB and their drug resistance (DR) profiles in Hunan province are still lacking. Therefore, this study retrospectively collected the records of patients with EPTB in Hunan Chest Hospital from 2013 to 2021 and analyzed their epidemiological and DR characteristics to provide evidence for the prevention and treatment of EPTB in Hunan province. It is worth mentioning that the present study provided a comprehensive view of the DR characteristics of EPTB and compared them between the subtypes of EPTB with large samples, which has never been reported.

## Materials and methods

### Data sources and collection

We retrospectively collected the records of inpatients with EPTB in Hunan Chest Hospital from 1 January 2013, to 31 December 2021. Hunan Chest Hospital is designated as a provincial institute and clinical center for TB, in addition to being a hospital that delivers treatments for cardiac diseases and thoracic tumors, enabling 1,600 patient hospitalizations. This hospital provides tertiary care for TB patients from Hunan province and parts of surrounding regions. It also accepts referrals for severe TB cases from other regions of China. In addition, operating under the supervision of the hospital authority, this hospital ensures compliance with strict protocols for patient care.

The diagnosis and classification of EPTB in Hunan Chest Hospital followed the national guidelines issued by the National Health and Family Planning Commission in China (11, 12). The inclusion criteria for the subjects were as follows: (1) inpatients diagnosed with EPTB, including EPTB combined with PTB (EPTB-PTB) and (2) EPTB patients with *Mycobacterium tuberculosis* isolates. The exclusion criteria included the following: (1) patients who lived in other provinces, (2) patients with incomplete case data; and (3) patients with repeated admissions within 1 year. We collected data regarding the demographic and clinical variables from electronic patient records using a standardized pretested questionnaire, including sex, age, occupation, place of residence, diagnosis, and drug susceptibility information. All sensitive patient information was removed before analysis.

We classified the subtypes of EPTB into nine types: lymphatic TB, musculoskeletal TB, pharyngeal/laryngeal TB, genitourinary TB, abdominal TB, tuberculous meningitis, tuberculous pericarditis, multiple EPTB, and other EPTB. Multiple EPTB refers to cases where patients had more than one subtype of EPTB, not limited to the former seven specified subtypes. Other EPTB denotes cases where patients did not exhibit any of the former eight specified subtypes of EPTB.

For the purpose of analyzing the drug susceptibility profiles of the patients, we excluded isolates from the same body sites within a month from an individual. Comparisons of DR rates among groups were made using individuals' first *M. tuberculosis* isolates.

### Culture and drug susceptibility testing

Laboratory examination was performed in the clinical laboratory of Hunan Chest Hospital, Changsha, China, a province-level reference laboratory specializing in mycobacterium detection and equipped with a full set of mycobacterium-detection facilities. Clinical specimens, including ascites, puncture fluid, pus, feces, and tissues, were collected from TB patients and treated with a 1% NaOH, 0.73% sodium citrate, and 0.25% N-acetyl-L-cysteine (NALC) solution for 15–20 min. For large tissue samples, such as surgically removed tuberculous lymph nodes and necrotic bones or muscles, small pieces of tissue were prepared and then treated with a 1% NaOH, 0.73% citric acid, and 0.25% NALC solution. According to the relevant guidelines, liquid cultures were carried out in a Bactec MGIT 960 instrument (Becton, Dickinson, and Company; Cockeysville, MD, USA). Solid cultures were performed by inoculating 200 μL of the decontaminated pellets onto the Lowenstein-Jensen (L-J) medium, then incubating at 37°C for up to 70 days. All *M. tuberculosis* isolates were validated using a growth test on a p-nitrobenzoic acid-containing medium (Baso, Zhuhai, China) or an MBP 64 antigen detection kit (Genesis, Hangzhou, China).

The *M. tuberculosis* isolates were also used for drug susceptibility testing using the Bactec MGIT 960 instrument. All steps were performed by trained and specialized staff in a biosafety cabinet following the relevant guidelines. The reference strain H37Rv was used for quality control once a month or for each new batch of susceptibility kits. The critical concentrations of MGIT 960 were 1.0 μg/mL for streptomycin (STR), 0.2 μg/mL for isoniazid (INH), 1.0 μg/mL for rifampicin (RFP), and 5.0 μg/mL for ethambutol (EMB) (13).

### Statistical analysis

Statistical data analysis was conducted using SPSS (version 21.0, SPSS Inc., Chicago, IL, United States). Descriptive statistical methods were used to present the distribution characteristics of EPTB. The Chi-squared or Mann–Whitney U tests evaluated categorical variables' associations. The odds ratio (OR) with a 95% confidence interval (CI) was determined using logistic regression models. A multivariable logistic regression model was used to identify factors associated with drug resistance. The statistical significance was established at a *P* < 0.05. We conducted the map drawing using R-Studio software (version 4.2.2).

## Results

### The overall epidemiology of EPTB

From 2013 to 2021, 1,403 patients with EPTB, whether concurrent with or without PTB, were identified at Hunan Chest Hospital. Of these patients, 79 were from other provinces, and therefore, we finally included 1,324 cases in our statistical analysis.

**Figure 1:**
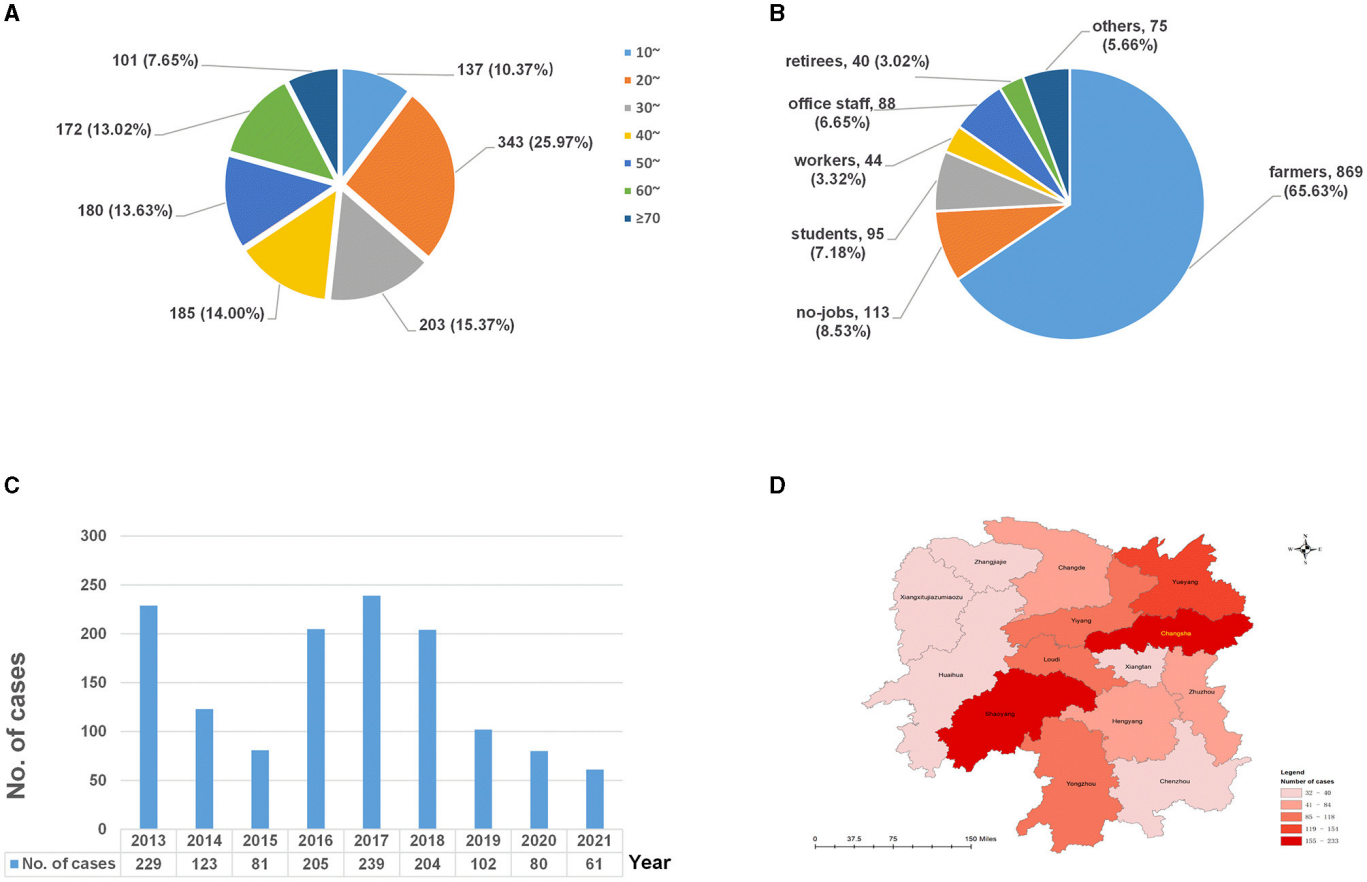
The distributions of extrapulmonary tuberculosis in Hunan, China, from 2013 to 2021. **(A)** Age distribution; **(B)** occupational distribution; **(C)** temporal distribution; and **(D)** spatial distribution.

Of the 1,324 cases, 635 (47.96%) were diagnosed exclusively with EPTB, while 689 (52.04%) were diagnosed with EPTB-PTB; 887 were male individuals and 437 were female individuals, resulting in a male-to-female sex ratio of 2.03. The age of the patients ranged from 3 to 87 years, with a median age of 38 (25%−75%: 25–56) years. The 20–29-year-old group accounted for most EPTB cases (25.91%, 343/1,324), followed by the ≥60-year-old group (20.62%, 273/1,324) (Figure 1A). Since there were only three cases under the age of 10 years, the age group of 0–9 years was not included in the analysis that segmented data by age groups.

In terms of occupational distribution (Figure 1B), the patients were mainly farmers, accounting for 65.63% (869/1,324), followed by the unemployed, who represented 8.53% (113/1,324), and students, who made up 7.18% (95/1,324) of the total cases.

As shown in Figure 1C, the number of EPTB patients was higher in 2013 and 2016–2018 (peaked in 2017) and showed a decreasing trend between 2019 and 2021.

In terms of the regional distribution (Figure 1D), EPTB in Hunan province was mainly distributed in Changsha (17.60%, 233/1,324), Shaoyang (15.94%, 211/1,324), and Yueyang (11.63%, 154/1,324), accounting for 45.17% of all cases, while cases from Zhangjiajie (2.64%, 35/1,324) and Xiangxi (2.42%, 32/1,324) were less.

### Distributions of EPTB subtypes

Lymphatic TB was the most prevalent subtype (29.83%, 395/1,324), followed by multiple EPTB (20.85%, 276/1,324) and musculoskeletal TB (14.65%, 194/1,324) (Table 1). We then compared the subtype distribution difference between exclusively EPTB cases and those with concurrent PTB and found it was statistically different (χ^2^= 235.049, *P* = 0.000). Musculoskeletal TB, genitourinary TB, and other forms of EPTB were more likely to present as exclusively EPTB, while lymphatic TB and pharyngeal/laryngeal TB were more likely to occur concurrently with PTB (Table 1).

**Table 1:** Distribution differences of subtypes between EPTB and EPTB-PTB.

**Subtypes of EPTB**	**No. of cases with exclusively EPTB (%)**	**Number of cases with EPTB-PTB (%)**	**Total cases (%)**	***χ^2^***	***P***
Lymphatic TB	151 (23.78)^a^	244 (35.41)^b^	395 (29.83)	235.049	0.000
Multiple EPTB	127 (20.00)^a^	149 (21.63)^a^	276 (20.85)		
Musculoskeletal TB	146 (22.99)^a^	48 (6.97)^b^	194 (14.65)		
Pharyngeal/laryngeal TB	3 (0.47)^a^	122 (17.71)^b^	125 (9.44)		
Genitourinary TB	78 (12.28)^a^	19 (2.76)^b^	97 (7.33)		
Abdominal TB	43 (6.77)^a^	39 (5.66)^a^	82 (6.19)		
Tuberculous meningitis	40 (6.30)^a^	31 (4.50)^a^	71 (5.36)		
Tuberculous pericarditis	19 (2.99)^a^	29 (4.21)^a^	48 (3.63)		
Other EPTB	28 (4.41)^a^	8 (1.16)^b^	36 (2.72)		
Total	635 (47.96)	689 (52.04)	1,324 (100.00)		

TB, tuberculosis; EPTB, extrapulmonary tuberculosis; EPTB-PTB, EPTB patients concurrent with PTB. Multiple EPTB refers to cases where patients have more than one subtype of EPTB, not limited to the specific subtypes listed in this table. Other EPTB refers to cases where patients did not have the specific subtypes of EPTB listed in this table, and they can also be categorized into exclusively EPTB and EPTB-PTB. The superscripted letters (number^a/b^) are used to show the results of one-by-one comparison using the Bonferroni method of one subtype. Both groups with the letter a mean that no significance was found. In contrast, there was statistical significance between the two groups.

We also analyzed the EPTB subtype distribution differences among various age groups using an R^*^C chi-squared test, which yielded statistically significant results (χ^2^= 136.58, *P* = 0.000) (Table 2). A further one-by-one comparison revealed that multiple EPTB, pharyngeal/laryngeal TB, abdominal TB, and tuberculous meningitis showed consistent patterns among all age groups, while other subtypes of EPTB showed their age-specific prevalence. For instance, lymphatic TB was most common in the 20-year age group, while genitourinary TB was most common in individuals in their 40s, followed by the 30-year age group; musculoskeletal TB was most common in the 60- and 20-year age groups; tuberculous pericarditis showed a sudden increase in the 60-year age group and peaked in the 70-year-old group. Other EPTB subtypes were most common in the 70-year age group. The results are shown in Table 2.

**Table 2:** Age distribution of EPTB subtypes.

**EPTB subtypes**	**No. of cases in each age group (years) (%)**							**Total**
	**10**~	**20**~	**30**~	**40**~	**50**~	**60**~	≥**70**	
Lymphatic TB	49 (12.5)^a, b^	132 (33.6)^b^	66 (16.8)^a, b, c^	38 (9.7)^c^	48 (12.2)^a, b, c^	38 (9.7)^a, c^	22 (5.6)^a, c^	393
Multiple EPTB	38 (13.8)^a^	82 (29.7)^a^	39 (14.1)^a^	40 (14.5)^a^	32 (11.6)^a^	25 (9.1)^a^	20 (7.2)^a^	276
Musculoskeletal TB	10 (5.2)^a^	37 (19.2)^a^	26 (13.5)^a^	29 (15.0)^a, b^	28 (14.5)^a, b^	44 (22.8)^b^	19 (9.8)^a, b^	193
Pharyngeal/laryngeal TB	7 (5.6)^a^	29 (23.2)^a^	21 (16.8)^a^	21 (16.8)^a^	28 (22.4)^a^	12 (9.6)^a^	7 (5.6)^a^	125
Genitourinary TB	7 (7.2)^a, b^	11 (11.3)^b^	20 (20.6)^a^	23 (23.7)^a^	17 (17.5)^a, b^	15 (15.5)^a, b^	4 (4.1)^a, b^	97
Abdominal TB	12 (14.6)^a^	23 (28.0)^a^	14 (17.1)^a^	13 (15.9)^a^	9 (11.0)^a^	9 (11.0)^a^	2 (2.4)^a^	82
Tuberculous meningitis	8 (11.3)^a^	18 (25.4)^a^	8 (11.3)^a^	11 (15.5)^a^	8 (11.3)^a^	1 3(18.3)^a^	5 (7.0)^a^	71
Tuberculous pericarditis	4 (8.3)^a^	6 (12.5)^a^	5 (10.4)^a^	4 (8.3)^a^	4 (8.3)^a^	11 (22.9)^a, b^	14 (29.2)^b^	48
Other EPTB	2 (5.6)^a, b^	5 (13.9)^b^	4 (11.1)^a, b^	6 (16.7)^a, b^	6 (16.7)^a, b^	5 (13.9)^a, b^	8 (22.2)^a^	36
Total	137 (10.4)	343 (26.0)	203 (15.4)	185 (14.0)	180 (13.6)	172 (13.0)	101 (7.6)	1,321

TB, tuberculosis; EPTB, extrapulmonary tuberculosis. Multiple EPTB refers to cases where patients have more than one subtype of EPTB, not limited to the specific subtypes listed in this table. Other EPTB refers to cases where patients did not have the specific subtypes of EPTB listed in this table, and they can also be categorized into exclusively EPTB and EPTB-PTB. The superscripted letters (number^a/b^) are used to show the results of one-by-one comparison using the Bonferroni method for one subtype of EPTB. No significance was found in any two age groups with the letters a or b. In contrast, there was statistical significance between the groups.

**Figure 2:**
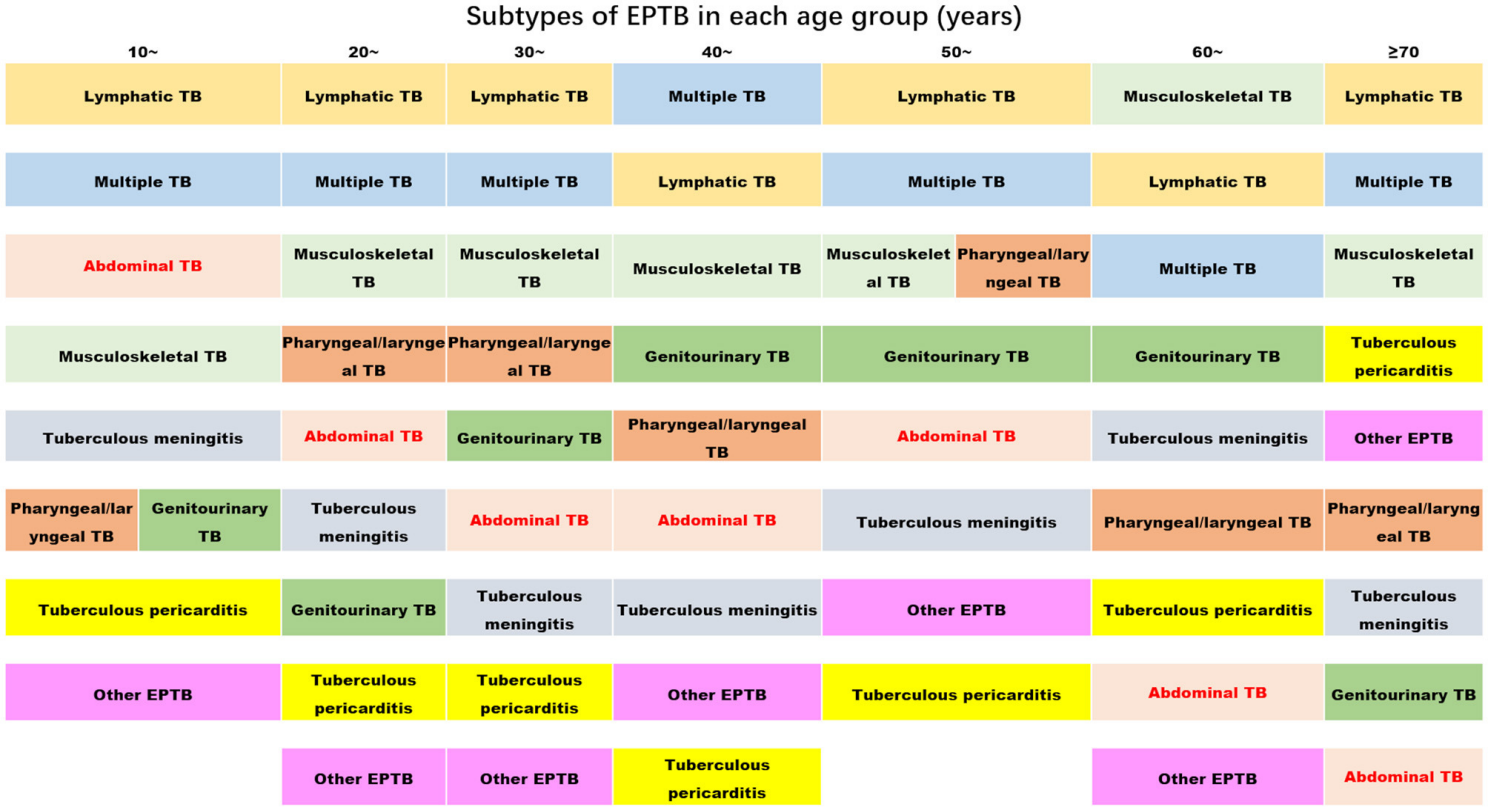
The ranks of the proportion of subtypes of extrapulmonary tuberculosis in each age group (the proportion decreases from top to bottom) (TB, tuberculosis; EPTB, extrapulmonary tuberculosis. Multiple EPTB means that patients have more than one subtype of EPTB, not limited to the specific subtypes listed in this figure. Other EPTB means that patients did not have the specific subtypes of EPTB listed in this figure).

Figure 2 clearly displays the predominant subtypes of EPTB across various age groups: Lymphatic TB showed the highest rate of prevalence across all age groups, with a slight decrease in the 40-year age group. Multiple EPTB was consistently the second most common subtype, except in the 40-year age group, where it ranked first, and in the 60-year age group, where it ranked third. Musculoskeletal TB ranked third across all groups but moved up to the first rank in the 60-year age group.

### Drug resistance in patients with EPTB

#### The summary of drug resistance

Between 2013 and 2021, from a total of 1,324 isolates, resistance to specific drugs was observed as follows: 164 isolates (12.39%) were resistant to RFP, 276 isolates (20.85%) to INH, 66 isolates (4.95%) to EMB, and 215 isolates (16.24%) to STR. Additionally, 148 isolates (11.18%) exhibited MDR (resistant to at least INH and RFP). Furthermore, 59 isolates (4.46%) were poly-DR (resistant to more than one drug but not MDR), and 126 (9.52%) were mono-DR (resistant to only one drug; mono-RFP, 11 isolates; mono-INH, 74 isolates; mono-STR, 40 isolates; and mono-EMB, 1 isolate). A total of 333 (25.23%) isolates displayed total DR (resistant to any of RFP, INH, EMB, and STR).

#### Temporal trend of drug resistance in EPTB

**Figure 3:**
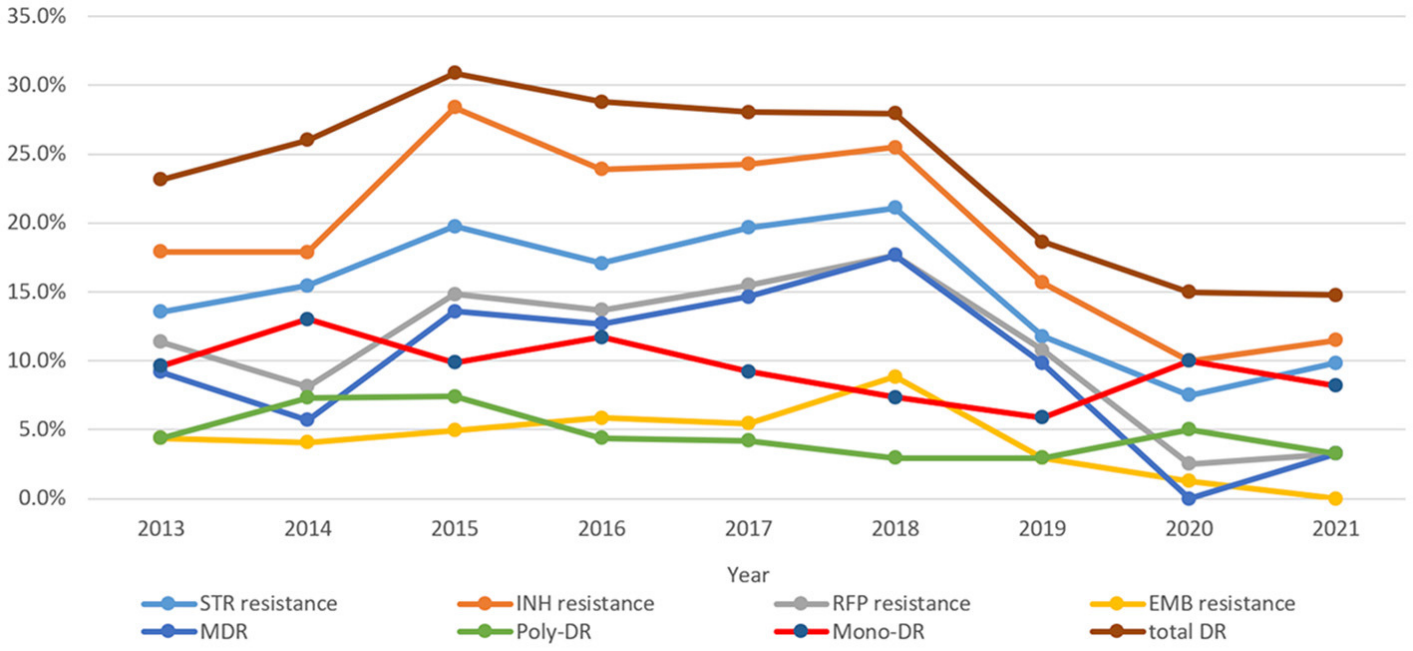
Drug resistance trends of extrapulmonary tuberculosis during 2013–2021 (STR, streptomycin; INH, isoniazid; RFP, rifampicin; EMB, ethambutol; MDR, multidrug resistance; total DR, total drug resistance, and it means that the isolates were resistant to any of RFP, INH, EMB, and/or STR).

As shown in Figure 3, the trends of resistance rates of three anti-TB drugs (RFP, INH, and STR), as well as total DR, poly-DR, and MDR, showed similar patterns over time. The INH resistance rates were the highest among all DR types over the course of 9 years. The rates of INH resistance, STR resistance, RFP resistance, and MDR remained high from 2015 to 2018; however, they showed downward trends in 2019–2020 and then increased in 2021. The rates of mono-DR and poly-DR both peaked in 2014 and 2020. The EMB resistance rate peaked in 2018. Comparison results for each of the eight DR rate differences among years showed that the STR resistance, INH resistance, RR/MDR, and MDR were significantly different between the years (χ^2^were 15.533, 20.770, 22.444, and 31.184, with *P-*values of 0.05, 0.008, 0.004, and 0.000, respectively) (Supplementary Table 1).

#### Age-specific drug resistance in EPTB

**Figure 4:**
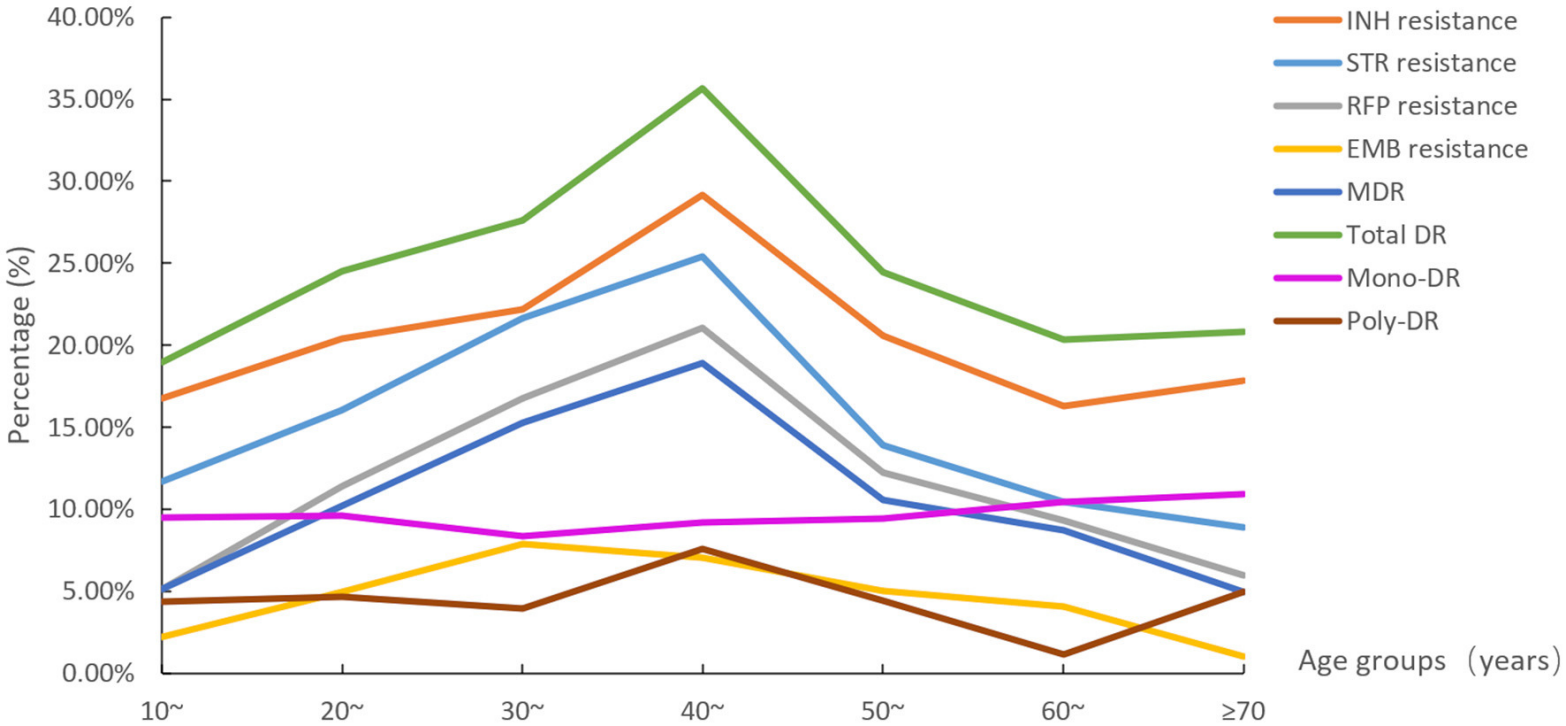
The eight drug resistance rate distributions among age groups (STR, streptomycin; INH, isoniazid; RFP, rifampicin; EMB, ethambutol; MDR, multidrug resistance; total DR, total drug resistance, and it means the isolates were resistant to any of RFP, INH, EMB, and/or STR).

The eight DR rate distributions among age groups are shown in Figure 4. The 40–49 age group had the highest resistance rates of the six types (except EMB resistance and mono-DR). The rates of STR resistance, RFP resistance, MDR, and total DR differed among all age groups (Supplementary Table 1).

### Drug resistance differences among subtypes of EPTB

We also compared the differences in each DR rate between exclusively EPTB cases and those with EPTB concurrent with PTB. Our analysis revealed that the rate of poly-DR was notably higher in exclusively EPTB cases compared to EPTB-PTB cases (5.98% vs. 3.05%) (Supplementary Table 1).

**Figure 5:**
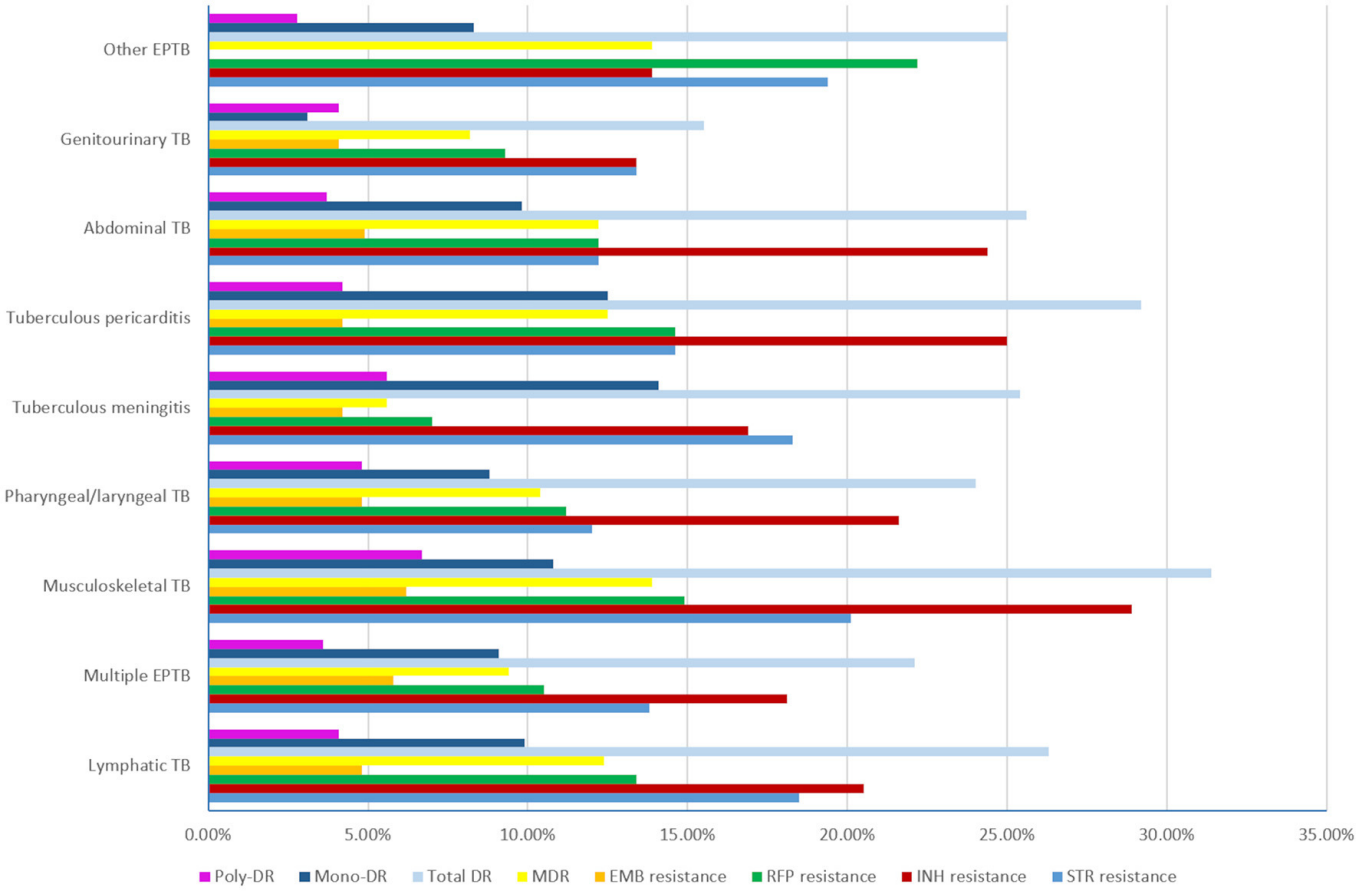
The drug resistance rates of the nine subtypes of extrapulmonary tuberculosis (TB, tuberculosis; EPTB, extrapulmonary tuberculosis; STR, streptomycin; INH, isoniazid; RFP, rifampicin; EMB, ethambutol; MDR, multidrug resistance; total DR, total drug resistance, and it means that the isolates were resistant to any of RFP, INH, EMB, and STR. Multiple EPTB means that patients have more than one subtype of EPTB, not limited to the specific subtypes listed in this figure. Other EPTB means that patients did not have the specific subtypes of EPTB listed in this figure).

**Figure 6:**
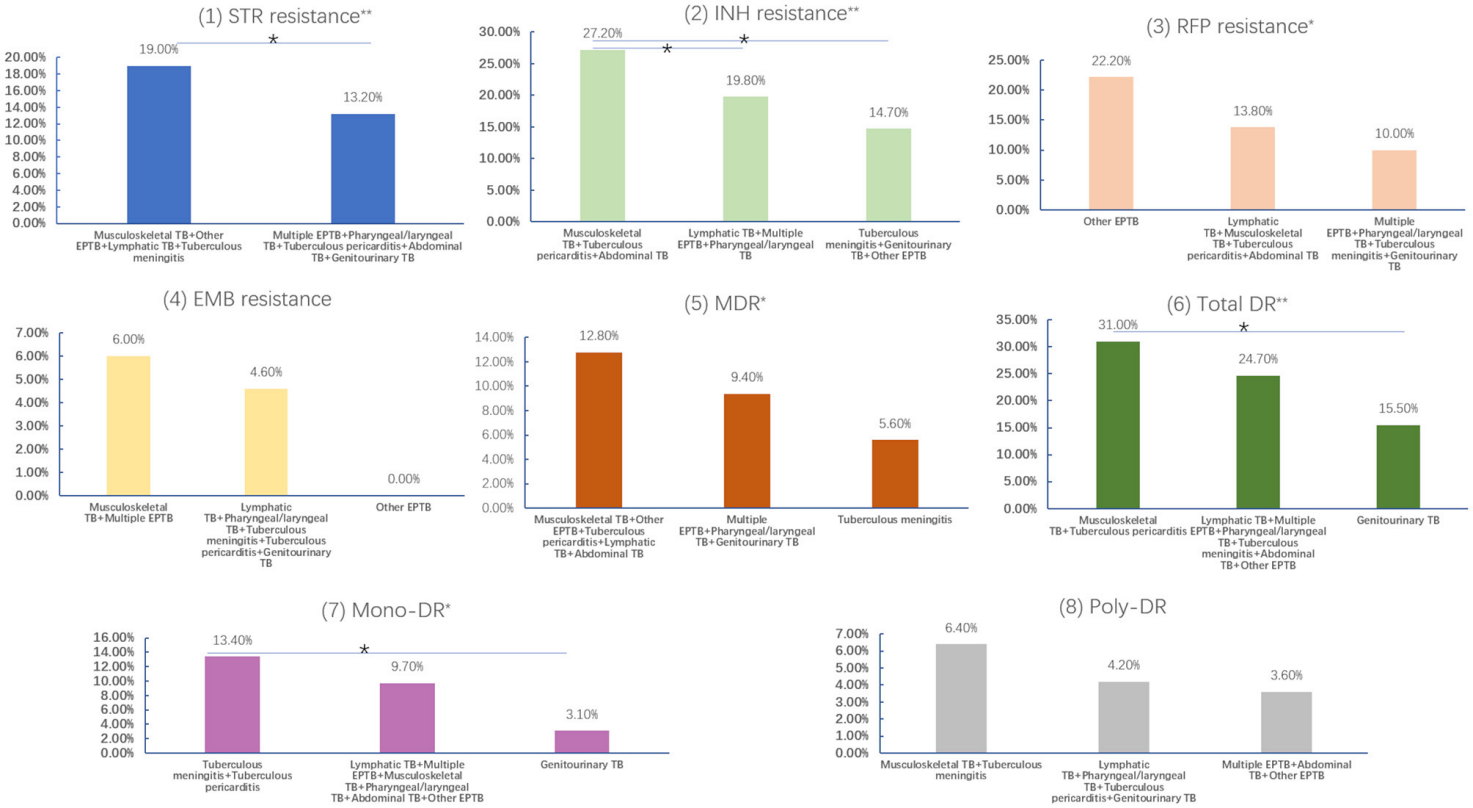
Drug resistance comparisons between different subtypes of extrapulmonary tuberculosis. (1) STR resistance; (2) INH resistance; (3) RFP resistance; (4) EMB resistance; (5) MDR; (6) Total DR; (7) mono-DR; (8) poly-DR (*with a line means that the drug resistance rates between two groups were significantly different by one-by-one comparison [Bonferroni test]. ^*^ and ^**^ after the subtitles means the resistance differences were statistically significant between/among groups, ^*^means *P* < 0.05, ^**^means *P* < 0.01. TB, tuberculosis; EPTB, extrapulmonary tuberculosis; STR, streptomycin; INH, isoniazid; RFP, rifampicin; EMB, ethambutol; MDR, multidrug resistance; total DR, total drug resistance, and it means that the isolates were resistant to any of RFP, INH, EMB, and/or STR. Multiple EPTB means that patients have more than one subtype of EPTB, not limited to the specific subtypes listed in this figure. Other EPTB means that patients did not have the specific subtypes of EPTB listed in this figure).

The rates of eight DR types for each EPTB subtype are shown in Figure 5. We found that musculoskeletal TB had the highest rates of total DR (31.40%), INH resistance (28.90%), STR resistance (20.10%), EMB resistance (6.20%), and MDR (13.90%) similar to that of other EPTB. and poly-DR (6.70%). The other EPTB type had the highest rate of RFP resistance (22.20%), and tuberculous meningitis had the highest mono-DR rate (14.10%); while pharyngeal/laryngeal TB had the lowest STR resistance rate (12.00%), tuberculous meningitis had the lowest RR (7.00%) and MDR (5.60%) rates, genitourinary TB had the lowest rates of INH resistance (13.40%), total DR (15.50%) and mono-DR (3.10%), and other EPTB had the lowest rates of EMB resistance (0.00%) and poly-DR (2.80%). However, no statistical significance was found when comparing each type of resistance among the nine sub-EPTB types (Supplementary Table 1). We then combined the groups with similar DR rates and compared them again. The results indicated significant differences in the six DR types between or among the groups, while two types (EMB resistance and poly-DR) did not show such variation (Figure 6).

### Drug resistance differences in region, sex, and occupation

Based on the locations of the 14 cities in Hunan province, we divided Hunan into five areas: east (Changsha, Xiangtan, and Zhuzhou), south (Hengyang, Yongzhou, and Chenzhou), west (Zhangjiajie, Xiangxi, and Huaihua), north (Yueyang and Changde), and center (Loudi, Yiyang, and Shaoyang). Then, we compared the differences of eight types of resistance rates among the EPTB patients from the five areas and found that the INH, RFP, STR, and EMB resistance, MDR, total DR, and mono-DR showed significant differences. The west of Hunan had the highest resistance rates of seven types (except mono-DR). When compared between the male and female individuals, none of the types of resistance rates showed a difference. Furthermore, when compared between different occupations, RFP resistance, MDR, and poly-DR showed significant differences; the workers had the highest resistance rates of RFP resistance and MDR. The details are shown in Supplementary Table 1.

### Associated factors with rifampicin/multidrug resistance in EPTB using multivariable logistic regression

RR/MDR presents a significant challenge in controlling TB. Consequently, this section focuses on its risk factors. We included all factors that showed statistically significant associations with RR/MDR EPTB from the univariate analysis into a multivariable logistic regression model.

We found that, compared to the patients diagnosed in 2013, those diagnosed in 2018 had a higher likelihood of developing RR/MDR, while those diagnosed in 2020 showed a reduced risk. Compared to the EPTB patients in the younger age group of 10 years, the patients aged between 20 and 59 years were at a risk of developing RR/MDR. Workers were more likely to develop RR/MDR compared to retirees. Compared to the patients from the east of Hunan, EPTB patients from the south and west of Hunan were more likely to develop RR/MDR. Subtype comparison revealed that having a diagnosis of EPTP outside of the eight defined EPTB types increased the risk for RR/MDR using the combination of multiple EPTB, pharyngeal/laryngeal TB, tuberculous meningitis, and genitourinary TB as reference. The details are shown in Table 3.

**Table 3:** Associated factors with rifampicin/multidrug resistance in EPTB using multivariable logistic regression.

**Characteristics**	**RR/MDR EPTB (n, %)**		***P***	***OR* (95% *CI*)**
	**Yes**	**No**		
**Year of onset**				
2013	26 (11.35)	203 (88.65)	Reference	
2014	10 (8.13)	113 (91.87)	0.302	0.661 (0.301–1.451)
2015	12 (14.81)	69 (85.19)	0.445	1.355 (0.621–2.955)
2016	28 (13.66)	177 (86.34)	0.582	1.185 (0.647–2.174)
2017	37 (15.48)	202 (84.52)	0.398	1.279 (0.723–2.260)
2018	36 (17.65)	168 (82.35)	0.049^*^	1.782 (1.004–3.165)
2019	11 (10.78)	91 (89.22)	0.932	1.034 (0.476–2.249)
2020	2 (2.50)	78 (97.50)	0.030^*^	0.191 (0.043–0.850)
2021	2 (3.28)	59 (96.72)	0.107	0.292 (0.065–1.304)
**Age groups (years)**				
10~	7 (5.11)	130 (94.89)	Reference	
20~	39 (11.37)	304 (88.63)	0.023^*^	3.068 (1.166–8.071)
30~	34 (16.75)	169 (83.25)	0.002^**^	5.184 (1.854–14.494)
40~	39 (21.08)	146 (78.92)	0.000^**^	6.808 (2.436–19.030)
50~	22 (12.22)	158 (87.78)	0.026^*^	3.382 (1.158–9.876)
60~	16 (9.30)	156 (90.70)	0.072	2.748 (0.914–8.268)
≥70	6 (5.94)	95 (94.06)	0.410	1.722 (0.472–6.278)
**Career**				
Retirees	1 (2.50)	39 (97.50)	Reference	
Office staff	7 (7.95)	81 (92.05)	0.494	2.154 (0.239–19.418)
Students	8 (8.42)	87 (91.58)	0.112	6.217 (0.655–59.035)
No jobs	11 (9.73)	102 (90.27)	0.328	2.926 (0.340–25.152)
Farmers	114 (13.12)	755 (86.88)	0.192	3.898 (0.505–30.116)
Others	12 (16.00)	63 (84.00)	0.132	5.254 (0.608–45.389)
Workers	11 (25.00)	33 (75.00)	0.046^*^	9.110 (1.043–79.572)
**Region**				
East of Hunan (Changsha, Xiangtan, and Zhuzhou)	30 (8.98)	304 (91.02)	Reference	
South of Hunan (Hengyang, Yongzhou, and Chenzhou)	36 (16.22)	186 (83.78)	0.023^*^	1.881 (1.092–3.241)
West of Hunan (Zhangjiajie, xiangxi, and Huaihua)	25 (23.58)	81 (76.42)	0.001^**^	2.749 (1.479–5.111)
North of Hunan (Yueyang, Changde)	28 (11.86)	208 (88.14)	0.277	1.369 (0.778–2.410)
Center of Hunan (Loudi, Yiyang, Shaoyang)	45 (10.56)	381 (89.44)	0.462	1.210 (0.728–2.012)
**Subtypes of EPTB for RFP/MDR analysis**				
Other EPTB	8 (22.2)	28 (77.8)	0.006^**^	3.545 (1.432–8.778)
Lymphatic TB+Musculoskeletal TB+Tuberculous pericarditis+Abdominal TB	99 (13.77)	620 (86.23)	0.055	1.438 (0.993–2.084)
Multiple EPTB+Pharyngeal/laryngeal TB+Tuberculous meningitis+Genitourinary TB	57 (10.02)	512 (89.98)	Reference	

^*^P < 0.05, ^**^P < 0.01. TB, tuberculosis; EPTB, extrapulmonary tuberculosis. Multiple EPTB refers to cases where patients have more than one subtype of EPTB, not limited to the specific subtypes listed in this table. Other EPTB refers to cases where patients did not have the specific subtypes of EPTB listed in this table.

## Discussion

We conducted a detailed analysis of the epidemiological and DR characteristics of EPTB at a provincial hospital in Hunan province, which is located in South-Central China, from 2013 to 2021. This is the first report to reveal the epidemiology of EPTB in this area. The results from this analysis will provide valuable insights for the prevention and treatment of EPTB in Hunan, China, and potentially inform strategies in other areas.

In this study, the overall male-to-female ratio was 2.03, which was higher compared to the findings of Colombia (1.68) (14), Turkey (1.57) (15), and the Federation of Bosnia and Herzegovina (1.26) (16), while contrary to that from a national investigation in China (0.70) (6). In terms of age, EPTB occurred most frequently at the age of 20–29 years, similar to the findings of other studies from the Federation of Bosnia and Herzegovina (16) and Saudi Arabia (17), but it was not consistent with the national survey in China, which showed that the 45–54 year age group accounted for the highest proportion (21.00%) (6), and another study on the Polish population showed that most EPTB patients were aged 0–19 years (18). Although few studies reported that the older adults were the main population affected by EPTB, the present study found that the over-60-year-old age group accounted for 20.63% of EPTB patients and ranked second.

The present study found that the main occupation of EPTB cases was farming, followed by those with no job and students. The explanation for most EPTB patients being farmers is that Hunan is a large agricultural province, and most of the population is concentrated in rural areas. Farmers with severe EPTB usually go to the provincial TB hospital and seek treatments. For the EPTB patients without jobs, we speculated that these patients had suffered for a long time from the disease and could not go out for work, indicating that EPTB poses a great burden on the population's health and lives. Students in the present study accounted for 7.18% of the EPTB patients and ranked third among all occupations. Of the students, 52.6% of them had EPTB-PTB, suggesting the importance of PTB surveillance among those who mainly exhibit EPTB symptoms to prevent TB spread. We also found that 37.89% (36/95) and 27.37% (26/95) of EPTB students had lymphatic TB and multiple EPTB, respectively.

In terms of the temporal distribution of EPTB, we found that in 2013 and 2016–2018, the number of EPTB cases in Hunan province was high compared with that in 2014–2015 and 2019–2021. The high rate of prevalence in 2013 can be explained as follows: first, Hunan province implemented 10 health measures to benefit the people in 2013, including optimizing the New Rural Cooperative Medical Service (NRCMS) compensation policy, expanding the reimbursement scope, and improving the compensation level of hospitalization; the second was to provide free anti-TB treatment for patients with active TB (19). The low incidence in 2020 and 2021 was undoubtedly associated with the COVID-19 epidemic, affecting patients' ability to seek medical advice and hospitalization (20).

In the present study, 52.04% (689/1,324) of the EPTB patients had concurrent PTB, a proportion lower than that reported in a large-scale multicenter observational study in China (2011–2017) (62.56%, 127 005/202 998) (4) and a study from Shanghai (2015–2020) (59.81%, 2,086/3,488) (21). However, this percentage was significantly higher than that found in a national survey in China (2020–2021) (13.21%, 222/1,681) (6). Among the subtypes of EPTB in our study, lymphatic TB was most prevalent (29.83%), followed by multiple EPTB (20.85%) and musculoskeletal TB (14.65%). In other studies from China, lymphatic TB and musculoskeletal TB were always among the top three subtypes of EPTB (6, 22). In addition, pleural TB or bronchial TB have also been listed as one of the top three forms of EPTB (4, 22, 23). It was noticed that since May 1, 2019, pleural TB or bronchial TB had been reported as PTB in the Tuberculosis Information Management System of China. We also defined the type of PTB with this rule in the present study, which could explain the differences in the most frequent types of EPTB between the present study and other studies in China.

Previous studies showed that PTB is usually combined with EPTB in cervical, hilar, and/or mediastinal lymph nodes, pharynxes/larynxes, bronchi, intestines, and meninges (4, 23). These disease characteristics were supported by our results, as we found that lymphatic TB (35.41%) and multiple EPTB (21.63%) were the top two forms of EPTB among EPTB-PTB patients. It was worth noting that nearly all the pharyngeal/laryngeal TB (98.4%, 123/125) cases were concurrent with PTB. As the pharynx/larynx is located in a special site connected to the respiratory and digestive systems, as well as the oral cavity, tuberculous pharynx/larynx may be caused by two routines: first, through direct bacterial invasion of the pharynx/larynx; second, through the bronchial transmission of advanced TB. Thus, due to the high prevalence of co-occurrence of pharyngeal/laryngeal TB and PTB in Hunan province, the pathogenesis of pharyngeal/laryngeal TB can be primarily attributed to PTB. The prevalence of pharyngeal/laryngeal TB was higher in Hunan than in Beijing (22) and Zhejiang (21) in China. One explanation may be that chewing betel quid (24) and eating hot and spicy fried foods are common in Hunan. These foods could cause long-term adverse oral irritation and increase the incidence of pharyngeal/laryngeal TB in PTB patients. Chewing betel quid is a known risk factor for oral cancer (25, 26) and oral submucous fibrosis (27, 28), and it affects the oral microbiota (24). The present study suggests that more studies are needed to confirm the association between pharyngeal/laryngeal TB and betel quid chewing.

We found that 16.73% (221/1,321) of the EPTB patients had more than one TB lesion (classified as multiple EPTB). Both the simple EPTB and EPTB-PTB patients showed similar frequencies of multiple EPTB, which may be explained by the fact that most of the EPTB patients in the present study were farmers, and their relatively low income led to delays in treatment and subsequently resulted in disease progression. On the other hand, the prevalence of multiple EPTB reflects the seriousness of the TB epidemic in Hunan, and more attention should be paid to controlling TB in this area.

Hunan is one of the provinces in China with a high burden of DR-TB (29). The proportions of DR-TB among all patients and previously treated patients in Hunan were 10.5% and 28.8%, respectively (30). In this study, among the EPTB patients, the total DR-TB accounted for 25.23%, which was close to the rate (28.8%) of previously treated patients (30). RFP is a key drug for TB treatment, and the incidence of RR/MDR is an important indicator of the severity of local DR-TB. We found that the frequency of RR/MDR of EPTB was 12.39%, which was more than twice the national level in new TB cases in China (5.71%) (31). We found that INH had the highest resistance rate (20.85%) among the four tested anti-TB drugs, which is higher than that found among the new cases (16.0%) in a national drug surveillance study in China (31). The patients in this study were from a provincial DR-TB-designated hospital. Many DR-TB EPTB patients from different cities in Hunan province were transferred to this hospital, which may overestimate the DR level in the area. Nonetheless, the severe DR of EPTB should still attract the attention of clinicians and public health departments and hint that more accurate diagnosis tools and special treatment regimens for EPTB should be developed, screened, and evaluated.

For the temporal trends of the resistance rates during 2013–2021, we found that the rates of INH resistance, STR resistance, RFP resistance, MDR, and total DR remained high from 2015 to 2018, and most peaked in 2015 and 2018. In 2015, only 81 EPTB patients were included. The high DR rates may be due to the small sample size. In 2018, EPTB patients showed high DR rates, which may be attributed to the promulgation of a TB management policy in Hunan province in 2018 (32). According to the “13th Five-Year Plan” for TB prevention and control in Hunan province, each county and city was required to set up one to two TB-designated medical institutions. All 14 cities (prefectures) in Hunan province were required to establish hospitals for the diagnosis and treatment of DR-TB, ensuring that DR-TB patients could access medical care locally. Thus, many DR-EPTB patients were transferred to our facility, a provincial DR-TB-designated hospital, which led to the peak of many types of resistance rates in 2018.

The analysis focusing on age, EPTB subtype, region, occupation, and sex-specific DR showed that individuals in the 40–49-year-old age group, those with musculoskeletal TB, patients from the west of Hunan, and workers faced the most severe situations of DR. In the present study, we only explored the risk factors for RR/MDR EPTB and found that the risk factors included being aged 20–59 years, being a worker, living in the west and south of Hunan, and having forms of EPTB, other than the eight defined EPTB types. These were compared to those aged 10–19 years, retirees, patients from the east of Hunana, and those with the mentioned combination of multiple EPTB subtypes. A previous study on unspecified TB patients showed that the MDR-TB detection rate was more common among individuals under the age of 51 years (14.1%) than those over the age of 50 years (9.3%) (33), which was not in line with our results, suggesting that EPTB exhibits unique resistance characteristics. In Hunan, more EPTB cases were reported in the northeast and southwest regions, particularly in the cities of Changsha, Shaoyang, and Yueyang, compared to other areas/cities in Hunan. Conversely, the west and south of Hunan showed higher rates of RR/MDR EPTB compared to the east of Hunan (including Changsha). We speculate that the higher incidence of RR/MDR EPTB patients from the west and south of Hunan may be influenced by patients from these regions seeking treatment at the provincial hospital due to inadequate local medical care. This could potentially lead to an overestimation of the RR/MDR EPTB burden in these areas.

There are some limitations in this study: first, there was no available information on the treatment history, outcomes, or co-morbidities of TB patients; thus, we could not comprehensively reflect the epidemiology of EPTB. Second, we only included the inpatients from the Hunan Provincial Chest Hospital. Given that inpatients with severe symptoms or DR-TB are more likely to be hospitalized, this selection bias could result in an overestimation of the prevalence of multiple EPTB and DR-EPTB.

## Conclusion

In conclusion, this study provides insights into the epidemiologic and DR characteristics of EPTB in Hunan province, China. Our findings indicate that the most common forms of EPTB are lymphatic TB, multiple EPTB, and musculoskeletal TB. Notably, musculoskeletal TB and genitourinary TB usually present exclusively as EPTB, while lymphatic TB and pharyngeal/laryngeal TB often occur concurrently with PTB. EPTB cases enrolled in 2018, patients in the 20–59 year-old age group, workers, and patients from the south and west of Hunan province are at high risk of developing RR/MDR. The high DR rates among EPTB (especially for musculoskeletal TB) highlight the need for timely diagnosis, effective drug susceptibility testing, and the development of more effective regimens for EPTB, with a particular focus on musculoskeletal TB.

## Data availability statement

The original contributions presented in the study are included in the article/Supplementary material, further inquiries can be directed to the corresponding authors.

## Ethics statement

The studies involving humans were approved by the Human Ethics Committee of Hunan Chest Hospital. The studies were conducted in accordance with the local legislation and institutional requirements. Written informed consent for participation was not required from the participants or the participants' legal guardians/next of kin in accordance with the national legislation and institutional requirements.

## Author contributions

YY: Data curation, Formal analysis, Funding acquisition, Methodology, Validation, Writing – original draft. YX: Formal Analysis, Methodology, Validation, Writing – original draft. HL: Conceptualization, Data curation, Project administration, Resources, Supervision, Writing – review & editing. SY: Conceptualization, Data curation, Formal Analysis, Methodology, Writing – review & editing. ML: Conceptualization, Investigation, Resources, Writing – review & editing. BL: Investigation, Supervision, Validation, Writing – review & editing. DaX: Formal Analysis, Investigation, Methodology, Validation, Writing – review & editing. YW: Data curation, Formal analysis, Investigation, Methodology, Writing – review & editing. WL: Investigation, Methodology, Validation, Writing – review & editing. TF: Investigation, Methodology, Validation, Writing – review & editing. JL: Investigation, Methodology, Validation, Writing – review & editing. DoX: Investigation, Methodology, Validation, Writing – review & editing. XY: Project administration, Supervision, Validation, Writing – review & editing. KW: Project administration, Resources, Supervision, Validation, Writing – review & editing. YT: Investigation, Project administration, Resources, Supervision, Validation, Writing – review & editing. GL: Conceptualization, Data curation, Formal analysis, Investigation, Methodology, Project administration, Software, Validation, Writing – original draft, Writing – review & editing.
